# Sacubitril/Valsartan Improves Left Atrial and Ventricular Strain and Strain Rate in Patients with Heart Failure with Reduced Ejection Fraction

**DOI:** 10.3390/life13040995

**Published:** 2023-04-12

**Authors:** Pedro Garcia Brás, António Valentim Gonçalves, Luísa Moura Branco, Rita Ilhão Moreira, Tiago Pereira-da-Silva, Ana Galrinho, Ana Teresa Timóteo, Pedro Rio, Ana Leal, Fernanda Gameiro, Rui M. Soares, Rui Cruz Ferreira

**Affiliations:** 1 Hospital de Santa Marta, Centro Hospitalar Universitário de Lisboa Central, 1169-024 Lisbon, Portugal; 2 NOVA Medical School, Faculdade de Ciências Médicas (NMS|FCM), 1169-056 Lisbon, Portugal

**Keywords:** left atrial strain, longitudinal strain, radial strain, circumferential strain, speckle tracking echocardiography, sacubitril/valsartan, heart failure with reduced ejection fraction

## Abstract

Background: Data on the impact of sacubitril/valsartan (SV) therapy on phasic left atrial (LA) and left ventricular (LV) strain in heart failure with reduced ejection fraction (HFrEF) are limited. The aim of this study was to evaluate changes in two-dimensional speckle tracking (2D-STE) parameters with SV therapy in HFrEF patients. Methods: Prospective evaluation of HFrEF patients receiving optimized medical therapy. Two-dimensional speckle tracking (2D-STE) parameters were assessed at baseline and after 6 months of SV therapy. LA strain and strain rate (SR) in reservoir, conduit, and contraction phases were compared with LV longitudinal, radial, and circumferential strain and SR and stratified according to heart rhythm and HFrEF etiology. Results: A total of 35 patients completed the 6-month follow-up, with a mean age of 59 ± 11 years, 40% in atrial fibrillation, 43% with ischemic etiology, and LVEF of 29 ± 6%. There were significant improvements in LA reservoir, conduit, and contractile strain and SR following SV therapy, particularly among patients in sinus rhythm. There were significant improvements in longitudinal, radial, and circumferential LV function indices. Conclusion: SV therapy in HFrEF was associated with improved longitudinal, radial, and circumferential function, particularly among patients in sinus rhythm. These findings can provide insights into the mechanisms underlying the improvement of cardiac function and help assess subclinical responses to the treatment.

## 1. Introduction

Sacubitril/valsartan, a neprilysin inhibitor and angiotensin II receptor blocker (ARB) combination, was shown to reduce cardiovascular mortality and heart failure (HF) hospitalization by 20% in comparison to enalapril in the PARADIGM-HF trial [1], in different age groups [2], in addition to improving the imbalance between the renin-angiotensin-aldosterone and natriuretic peptide systems [3].

Sacubitril/valsartan is currently recommended as a Class I alternative to angiotensin-converting enzyme inhibitors (ACEI) according to the most recent Heart Failure Guidelines for ambulatory patients with HF with reduced ejection fraction (HFrEF) [4] who continue to experience symptoms despite receiving optimal care, including ACEI (or an ARB, if they were unable to tolerate an ACEI), a beta-blocker (BB), and a mineralocorticoid receptor antagonist (MRA). Nevertheless, despite this recommendation and expanding indications, it has not been widely incorporated into clinical practice to the extent that was anticipated [5,6].

Previous observational and retrospective studies have demonstrated that sacubitril/valsartan induces early reverse remodeling in HFrEF patients, with a significant reduction in left ventricular (LV) dimensions, improvement in LV ejection fraction (LVEF)—both in patients with nonischemic and ischemic HFrEF—and improvement in diastolic function, systolic pulmonary artery pressure, mitral and tricuspid valve insufficiency [7,8,9,10]. LV reverse remodeling with sacubitril/valsartan therapy was described in HFrEF patients without diabetes versus diabetic patients [11]. However, sacubitril/valsartan was not associated with a reduced risk of ventricular tachyarrhythmias in chronic HFrEF patients [12].

Two-dimensional (2D) speckle tracking echocardiography (STE) is a novel tool used to assess the mechanical function of the left atrium (LA) and left ventricular (LV). The examination of LA myocardial deformation with longitudinal strain analysis can further detail LA phasic function, with greater sensitivity compared to volumetric measurements [13]. The three phases of the cardiac cycle—the reservoir phase in systole with LA filling, the conduit period in early diastole with passive LV filling, and the contractile (booster pump) phase with active contractility in late diastole—can be evaluated using strain analysis [14].

Regional LV mechanics in the longitudinal, radial, and circumferential directions can be accurately analyzed via two-dimensional STE [15], with subclinical strain impairment seen in all three directions among patients with cardiovascular risk factors [16]. Global longitudinal strain (GLS) is regarded as a sensitive and accurate indicator of cardiac remodelling and function, with current clinical application in the evaluation of ischemic heart disease [17], diastolic dysfunction, cardiomyopathies, and subclinical myocardial dysfunction in patients with valve disease or in patients undergoing chemotherapy [18].

Retrospective studies have demonstrated an improved LV GLS [19,20], LA reverse remodeling [21,22], and LA reservoir strain [23] with sacubitril/valsartan. Patients with significant reverse remodeling also had a decreased risk of cardiovascular death and HF hospitalization [23]. Prospective evidence on the effects of sacubitril/valsartan therapy on LA phasic strain and LV deformation parameters via STE is lacking. Further data could contribute to understanding the mechanism of reverse remodeling associated with this treatment and could be used in clinical practice as a prognostic indicator.

The objective of this study was to prospectively assess LA and LV reverse remodeling with strain imaging parameters before and after sacubitril/valsartan therapy in a real-world cohort of chronic HFrEF patients receiving optimal care medication. A sub-analysis regarding cardiac rhythm and heart failure etiology was also outlined.

## 2. Materials and Methods

### 2.1. Study Population

A prospective single-center analysis was performed from October 2017 to June 2018.

Ambulatory patients with chronic HF receiving an optimal standard of care therapyW, LVEF ≤40%, and New York Heart Association (NYHA) class II were asked to start sacubitril/valsartan therapy according to the current guidelines [24].

### 2.2. Definition of Chronic HF with Optimized Standard of Care Therapy

The optimized standard of care therapy for chronic HF was defined as:

1. Treatment for at least 6 months with the maximum tolerable doses of an ACEI (or ARB if appropriate), a BB, and an MRA;
2. Implantable cardioverter-defibrillator (ICD) and/or cardiac resynchronization therapy (CRT), if indicated;
3. Adequate care in accordance with appropriate guidelines for mitral regurgitation and coronary artery disease [24];
4. No anticipated therapy modifications for the ensuing 6 months;
5. The analysis was conducted prospectively from October 2017 to June 2018, and at that time, recommendations did not consider SGLT2 inhibitors to be a part of optimal HF therapy for non-diabetic patients [24].

### 2.3. Exclusion Criteria

1. Age under 18 years;
2. Cardiac surgery; ICD/CRT implantation; atrial fibrillation ablation; or percutaneous mitral regurgitation treatment in the previous 6 months;
3. Planned cardiac surgery; ICD/CRT implantation; atrial fibrillation ablation; or percutaneous mitral regurgitation treatment in the following 6 months;
4. Glomerular filtration rate (GFR) < 30 mL/min;
5. Baseline potassium values ≥ 5.5 mEq/L;
6. Child–Pugh class B or C.

### 2.4. Study Protocol

Every patient signed an informed consent form. Clinical, laboratory, and transthoracic echocardiography data were gathered in the week before starting treatment with sacubitril/valsartan. Sacubitril/valsartan was switched from an ACEI after the recommended 36 h washout time.

Sacubitril/valsartan therapy was started at 49/51 mg twice daily or 24/26 mg twice daily in patients receiving a dose of less than 10 mg/day of enalapril or equivalent.

With the exception of patients with a systolic blood pressure of less than 100 mmHg, symptomatic hypotension, hyperkalemia greater than 5.5 mEq/L, or a decrease in glomerular filtration rate to less than 60 mL/min as determined by the Cockcroft–Gault equation, the goal was to double the dose every 2 to 4 weeks to reach the target maintenance dose of 97/103 mg twice daily.

All patients were reassessed 6 months after starting sacubitril/valsartan, with reassessment including clinical evaluation, laboratory testing, and transthoracic echocardiography.

### 2.5. Transthoracic Echocardiogram

Using a GE Vivid E95 ultrasound system, a full TTE investigation with a frame rate of 50–60 frames/s was carried out at baseline and 6 months after sacubitril/valsartan.

Patients were lying in left lateral decubitus, and a 3.5 MHz transducer was used to conduct the investigation. All of the examinations were carried out by two cardiologists with echocardiography experience. Simpson’s biplane method was used to determine LVEF.

For the assessment, recommendations regarding LAVI, published by The European Association of Cardiovascular Imaging (EACVI), were followed using the biplane algorithm, which incorporates both the four-chamber and two-chamber apical views [14]. Patients were euvolemic at the time of the scan, in order to lessen the impact of loading conditions on volumetric measurement. Images were saved in cine loop format with three consecutive beats and uploaded to a workstation for further digital analysis using EchoPAC^®^ (version 202, GE Healthcare, Chicago, IL, USA).

**Figure 1:**
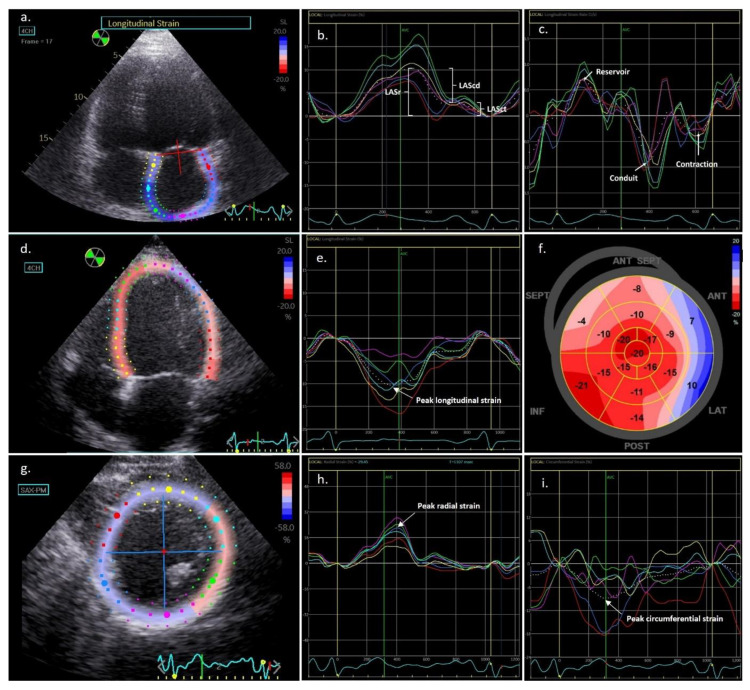
Left atrial and ventricular strain analysis via speckle tracking echocardiography. (**a**) Measurement of left atrial strain, semi-automatic contouring of the left atrial borders for speckle tracking; (**b**) Display of the left atrial deformation over a cardiac cycle starting at the QRS, with the 3 phases: reservoir (LASr), conduit (LAScd), and contractile (LASct) function; (**c**) Strain rate measurements in the reservoir, conduit, and contraction phases; (**d**) Measurement of left ventricular (LV) longitudinal strain, semi-automatic contouring of the endocardial borders for speckle tracking; (**e**) Display of the LV longitudinal strain over a cardiac cycle, with the arrow showing the peak longitudinal strain, (**f**) Global longitudinal strain bull’s eye plot in a patient with ischemic heart failure with reduced ejection fraction and basal antero-lateral hypokinesia; (**g**) Measurement of LV radial and circumferential strain with semi-automatic contouring; (**h**) Display of the LV radial strain over a cardiac cycle, with the arrow showing the peak longitudinal strain; (**i**) Display of the LV circumferential strain over a cardiac cycle, with the arrow showing the peak longitudinal strain.

Offline analysis of left atrial 2D STE data was conducted according to current recommendations [14]. The region of interest (ROI) width was briefly modified to the narrower atrium wall after manually tracing the LA endocardium (Figure 1). The LA endocardial and epicardial were extrapolated in parts of the LA wall discontinuities, such as areas corresponding to pulmonary veins, to achieve an accurate ROI. Six segments of the ROI were created, and these segments were examined using the strain software to generate a segmental longitudinal strain curve and a global LA strain.

Measures of LA deformation tracking were carried out using the R wave as a starting point (R-R gating). LA strain and strain rate values were registered in the reservoir, conduit, and contraction phases of the cardiac cycle (Figure 1). Contraction phase strain was only calculated among patients in sinus rhythm at the time of the scan.

Semiautomated speckle-based strain analyses in the conventional three short-axis views (LV basal, midventricular, and apical) and three apical views (LV four-chambers, two-chambers, and three-chambers) were performed to quantify the 2D LV deformation [15,25]. The reference point was placed at the start of the QRS complex, and the best cardiac cycle for each view was selected. By matching the epicardial border with the endocardial border, the ROI was modified to exclude the pericardium (Figure 1). In addition to the automated tracking detection in the program, the integrity of the tracking was verified visually and based on the accuracy of the strain curves. The ROI was revised as appropriate, and portions with continuously poor tracking were excluded. The peak systolic longitudinal strain and strain rate were calculated at the basal, midventricular, and apical levels and were then averaged into a global value for each direction. Peak systolic radial and peak systolic circumferential strain and strain rate were measured at the basal, midventricular, and apical levels. These measurements were then averaged to obtain a global value for each short-axis level and strain type. All strain readings were determined over the entire cardiac wall. The algorithm for calculating LV filling pressures in HFrEF patients with mitral inflow pulsed Doppler and mitral annulus Tissue Doppler Imaging [26] was used to grade the diastolic dysfunction.

### 2.6. Statistical Analysis

The Statistical Package for the Social Sciences (SPSS), version 23.0 for Windows, was used to conduct the statistical analysis. All mean estimates were shown as point estimates with a 95% confidence interval.

Descriptive statistics were reported as absolute frequency (number) and relative frequency for categorical variables (percentage). Continuous variables with normal distribution were presented as mean (and standard deviation), while those with abnormal distribution were presented as median (and interquartile range [IQR]).

The Kolmogorov–Smirnov test and visual histogram analysis were employed to verify normality. Pearson’s chi-squared test was used to compare categorical variables. Normally distributed variables before and after sacubitril/valsartan therapy were compared using the paired samples *t*-test, and non-normally distributed variables were compared using the Wilcoxon signed-rank test. The Mann–Whitney U test or Student’s *t*-test were used accordingly to compare continuous variables. Every statistical hypothesis test was performed with a significance threshold of α = 5%. 

A previous study investigated the impact of sacubitril-valsartan in LA reservoir strain and LV GLS in HFrEF patients [23]. This study enrolled 409 patients assessed with 1258 echocardiograms and showed a statistically significant improvement in LA reservoir strain from 11.4% (IQR 8.4–15.6%) to 15.9% (IQR: 11.5–21.4%) and in GLS from 10.2% (IQR 7.9–12.7%) to 13.9% (IQR 10.5–16.3%) within 6 months, which represented a 39.5% and a 36.3% improvement, respectively [23]. Based on these estimates and given that multiple parameters were assessed in our study, we opted to base our power sample estimation on the values for the LA reservoir strain and LV GLS. For a power of 80% at a 0.05 (α) significance, an n of 35 patients would be able to detect an improvement of 30% from a mean baseline LA reservoir strain of 11.5 ± 6.2% and from a mean baseline GLS of 7.0 ± 2.6%, using a paired samples *t*-test. Power and sample size calculations were performed using Stata^®^ Software Package, Version 17.0 (StataCorp LP, College Station, TX, USA).

Using a randomly chosen group of 20 patients, the intraobserver and interobserver variability for LA and LV 2D STE measures were evaluated. Measurements were repeated by the same observer after an interval of ≥1 week and by a second independent blinded observer. The intraclass correlation coefficient was used to measure reproducibility, with a good agreement designated as >0.80.

## 3. Results

### 3.1. Baseline Characteristics

**Figure 2:**
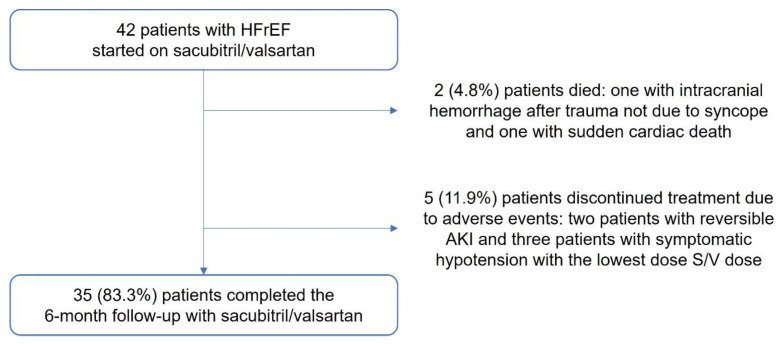
Study population flowchart. HFrEF, Heart failure with reduced ejection fraction; AKI, Acute kidney injury; S/V, Sacubitril/valsartan.

A total of 42 patients were enrolled in the trial. Out of these, 35 (83.3%) underwent the six-month follow-up with sacubitril/valsartan. Overall, 5 (11.9%) patients discontinued treatment due to adverse events (2 patients with reversible acute kidney injury and 3 patients with symptomatic hypotension with the lowest sacubitril/valsartan dose), and 2 (4.8%) patients died (one from intracranial hemorrhage following trauma not due to syncope and one with sudden cardiac death) (Figure 2).

The 35 patients successfully completed the six-month follow-up and the baseline characteristics are shown in Table 1. The mean age of participants was 58.6 ± 11.1, 82.9% male, and 42.9% had ischemic etiology. Patients were hospitalized due to worsening HF in the year before starting sacubitril/valsartan in 42.9% of cases, and significant symptomatology as indicated by a NYHA class III in 51.4% of cases. 94.3% of patients were taking an MRA, and all of them were taking an ACEI or ARB in conjunction with a BB.

In total, 31.4% were on SGLT2 inhibitors, and 85.6% of patients had an ICD, of which 20% had a CRT-D system. Three (8.6%) of the patients had previously had a transcatheter edge-to-edge mitral valve repair (MitraClip^®^, Abbott Vascular, Chicago, IL, USA).

### 3.2. Sacubitril/Valsartan Dose

Sacubitril/valsartan therapy was started at 24/26 mg twice daily among 18 (51.4%) patients and at 49/51 mg twice daily among 17 (48.6%) patients.

At six months, 40.0% were taking 97/103 mg twice daily, 28.6% were taking 24/26 mg twice daily, and 31.4% were taking 49/51 mg twice daily.

We discovered no significant differences in the percentage of the target BB dose (68.8 ± 28.6% vs. 70.6 ± 28.0%, *p* = 0.278), the dose of MRA (51.6 ± 19.0% vs. 53.2 ± 24.4%, *p* = 0.352), or the dose of the loop diuretic reported as furosemide equivalents (43.6 ± 27.6% vs. 39.1 ± 26.5%). No variations were found in the potassium levels at the beginning and end of treatment (4.5 ± 0.4 vs. 4.6 ± 0.4 mEq/L, *p* = 0.292).

### 3.3. Clinical Assessment

The 35 patients who finished the six-month sacubitril/valsartan course had a significantly improved NYHA class. A total of 24 patients (68.6%) improved one NYHA class, and 2 patients (5.7%) improved two classes, with 9 patients (25.7%) remaining in the same class. During the 6 months of sacubitril/valsartan therapy, no declines in NYHA class were observed, and only 3 (8.6%) patients stayed in class III. Only 3 (8.6%) patients experienced a cardiovascular hospitalization during the six-month follow-up period.

### 3.4. ECG Analysis

Atrial fibrillation (AF) was present in 14 (40.0%) patients at the time of the initial evaluation, with paroxysmal AF in 5 (14.3%) and permanent AF in 9 (25.7%) patients. At six months, there were no new cases of AF or catheter ablation in any patients.

### 3.5. Transthoracic Echocardiography Assessment

The outcomes of the TTE analysis are shown in Table 2. After 6 months of therapy, LV dimensions and atrial volumes were significantly reduced. There was no variation in the tricuspid annular plane systolic excursion. Diastolic dysfunction [26] was found in 14 (40%) patients after 6 months of sacubitril/valsartan medication compared to 22 (62.9%) patients with a grade of at least II at baseline. The left atrial volumes (51.5 ± 22.6 vs. 43.7 ± 15.8 mL/m^2^, *p* = 0.004) significantly decreased, despite there being no changes in the E/e’ ratio. Following treatment, there was a significant increase in LVEF (29.3 ± 6.4 vs. 35.2 ± 8.6%, *p* = 0.001), which was significantly higher in patients with a better NYHA class (29.7 ± 6.6% to 40.1 ± 7.7%, *p* = 0.001 vs. 28.6 ± 6.2% to 32.6 ± 8.9, *p* = 0.008; *p*-value [interaction] = 0.003).

#### 3.5.1. LA Strain Assessment

**Figure 3:**
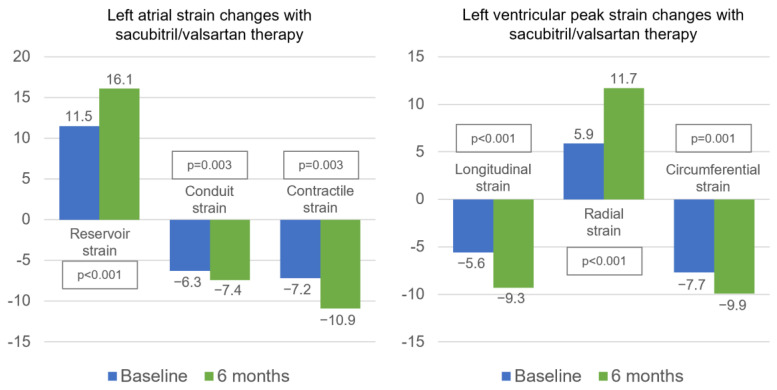
LA and LV strain changes after 6 months of sacubitril/valsartan therapy.

Table 2 and Figure 3 show the LA strain results at baseline and after 6 months of sacubitril/valsartan. After 6 months of treatment, there was a noticeable improvement in LA strain and strain rate in all phases of the cardiac cycle. Only patients in sinus rhythm during the scan (*n* = 26, 74.3%) were assessed for contractile strain and strain rate. Notably, patients who improved their NYHA class with treatment also experienced a statistically significant improvement in their LA reservoir phase strain (12.5 ± 6.2% to 18.1 ± 7.2%, *p* = 0.001 vs. 8.9 ± 5.4% to 11.0 ± 7.1%, *p* = 0.049; *p*-value [interaction] = 0.031) and reservoir strain rate (0.50 ± 0.21 s^−1^ to 0.71 ± 0.18 s^−1^, *p* < 0.001 vs. 0.46 ± 0.26 s^−1^ to 0.49 ± 0.25 s^−1^, *p* = 0.601; *p*-value [interaction] = 0.011).

#### 3.5.2. LV strain Assessment

After 6 months of therapy, GLS, peak systolic longitudinal strain, systolic strain rate, and diastolic strain rates all significantly improved (Table 2, Figure 3). Additionally, the peak radial strain, systolic strain rate, and early diastolic strain rate of the radial LV function were improved. Additionally, the circumferential strain, systolic strain rate, and late diastolic strain rates were all improved with the use of sacubitril/valsartan (Table 2, Figure 3). Only patients in sinus rhythm were assessed for LV late diastolic strain rate during the scan (*n* = 26, 74.3%). For LA and LV strain measurements, the intraclass correlation coefficient was 0.94 for intraobserver variability and 0.87 for interobserver variability.

#### 3.5.3. Sinus Rhythm versus Atrial Fibrillation Subanalysis

The baseline variations in the TTE parameters between patients with sinus rhythm and those with atrial fibrillation are illustrated in Supplementary Table S1. Patients in sinus rhythm showed a substantial improvement in LA reservoir strain, conduit strain, and reservoir strain rate with therapy in a subanalysis according to heart rhythm (Table 3). Patients in AF who were given sacubitril/valsartan showed a trend toward improvement, but there was no statistically significant improvement in LA deformation in this cohort. The strain and strain rate during contraction phases were not considered in this subanalysis.

Sacubitril/valsartan treatment improved the GLS, peak LV longitudinal strain, longitudinal systolic, and early diastolic strain rates in patients with sinus rhythm, while only peak longitudinal strain and early diastolic strain rate were significantly improved in patients in AF.

Peak LV radial strain significantly improved in both groups. However, systolic and early diastolic strain rates were improved only among patients in sinus rhythm. Only patients in sinus rhythm experienced an improvement in circumferential LV strain and strain rates (Table 3). Additionally, patients in sinus rhythm showed greater improvements following sacubitril/valsartan therapy than patients in AF regarding LA reservoir strain, LVEF, peak circumferential strain, and longitudinal systolic strain rate.

#### 3.5.4. Nonischemic versus Ischemic Etiology Subanalysis

Supplementary Table S2 shows the baseline variations in TTE parameters between patients with nonischemic and ischemic HFrEF. After 6 months of treatment with sacubitril/valsartan, there was a significant improvement in LA reservoir phase strain both in nonischemic and ischemic HFrEF patients. Patients with ischemic etiology showed an improved LA conduit phase strain, while patients with nonischemic improved contraction phase strain. Concerning LA strain rate, a significant improvement in strain rate in the LA reservoir and contraction phases was noted in patients with nonischemic etiology. Patients with ischemic HFrEF presented an improved conduit phase strain rate (Table 4). Regarding LV strain, both patients with nonischemic and ischemic etiology showed a significant improvement in peak longitudinal strain (especially patients with nonischemic etiology) and longitudinal systolic, early, and late diastolic strain rates. Both groups presented an improved peak circumferential strain, peak radial strain, and radial early diastolic strain rate after 6 months of treatment, and nonischemic HFrEF patients also showed an improved radial and circumferential systolic strain rate. Moreover, there was a greater improvement in peak longitudinal strain after undergoing sacubitril/valsartan therapy in patients with nonischemic etiology versus patients with ischemic etiology (Table 4). None of the groups demonstrated an improvement in circumferential early and diastolic strain rates or radial late diastolic strain rate.

## 4. Discussion

Our study’s key conclusion was that, after 6 months of sacubitril/valsartan therapy, patients with HFrEF demonstrated improvements in LA phasic strain, strain rates, and LV deformation indices, including longitudinal, radial, and circumferential strain and strain rates, particularly among patients in sinus rhythm, and independently of the cause of HFrEF. Our study also provided a summary of the individual phasic atrial function parameters as well as longitudinal, radial, and circumferential LV function STE parameters, stratifying them according to heart rhythm and HFrEF etiology, and evaluating their changes with sacubitril/valsartan treatment.

We believe that this is the first prospective study to evaluate the effects of sacubitril/valsartan on LA strain assessment in the various cardiac cycle phases, LV radial and circumferential strain, and longitudinal, radial, and circumferential systolic and diastolic strain rates. Sacubitril/valsartan therapy improved LVEF, GLS, and the majority of the STE LA and LV strain parameters in a group of highly symptomatic chronic HFrEF patients, as indicated by a NYHA class of at least III in 51.4% (compared to only 23.9% in the PARADIGM-HF) and by 42.9% experiencing hospitalizations due to worsening heart failure in the previous year.

The assessment of LA strain and strain rate with 2D STE has been increasingly used for an objective evaluation of LA function (reservoir, conduit, and contractile), as well as the assessment of LV diastolic function and the early detection of atrial dysfunction with prognostic significance in various pathological entities [27,28]. Notably, LA strain assessment has demonstrated a better sensitivity than LA volumes [13], and the results obtained with 2D STE are not directly comparable to those obtained via volumetric measures [28,29]. Additionally, STE parameters have shown good interobserver agreement between novice and experienced observers as well as excellent intraobserver reproducibility [30]. In our study, there was a 17.5% relative improvement in LA conduit strain and a 40% relative improvement in LA reservoir strain, regardless of the cause of HFrEF. Notably, sacubitril/valsartan treatment improved the LA deformation velocity as measured by strain rate, with the reservoir and conduit phases of the LA strain rate with a relative increase of 36.7% and 23.4%, respectively. This is consistent with a recent retrospective study by Moon et al. [23] that demonstrated improved LA reservoir strain within 6 months of sacubitril/valsartan therapy and established a link between left heart reverse remodeling and a reduced risk of major cardiovascular events, highlighting its potential as a prognostic indicator or as a possible surrogate of response to treatment. Furthermore, regardless of HFrEF etiology, our study’s findings support the clinical use of LA reservoir strain in HFrEF patients receiving sacubitril/valsartan therapy, as there was an association between clinical improvement (measured by an improved NYHA class) and an improved LA reservoir function.

Our results demonstrate a significant improvement in LA deformation with treatment in HFrEF patients with sinus rhythm and show a near-significant trend in patients with AF. Notably, patients in sinus rhythm showed a statistically significant improvement in LA reservoir strain compared to patients with atrial fibrillation (AF). This finding would be in keeping with the atrial structural remodeling and development of fibrosis in long-standing AF [31], particularly as the majority (64%) of AF patients in our cohort had permanent AF. However, this is merely hypothesis-generating, as our sample size is statistically insufficient to establish a direct subgroup link between heart rhythm and treatment response (see Limitations section). 

Although it is not currently as widely accepted, the evaluation of contractile phase strain (booster pump function) has been suggested as an alternative to the reservoir function [27]. It demonstrably had a good predictive value for elevated LV filling pressure [32] and is deserving of consideration in future studies [33]. With therapy, LA contraction strain in our sample showed a relative improvement of 51.4% and, for LA contraction strain rate, a relative improvement of 31.7%. However, as it can only be measured in patients with sinus rhythm during the scan, booster pump function was only examined in 74.3% of patients.

Prior research has demonstrated that sacubitril/valsartan promotes early LV reverse remodeling in chronic HFrEF patients via TTE examination [7,8,34], including GLS, LV twist, and rotation [20]. However, prospective information on radial and circumferential LV deformation following sacubitril/valsartan therapy is lacking. When compared to the EACVI NORRE research reference values, the LV longitudinal, radial, and circumferential strain levels in our chronic HFrEF group were significantly lower at baseline [25]. Peak LV longitudinal, radial, and circumferential deformation significantly increased within 6 months of sacubitril/valsartan therapy in both the ischemic and nonischemic HFrEF subgroups. While patients with nonischemic etiology showed a higher improvement in peak longitudinal strain compared to patients with ischemic etiology, our sample size was statistically underpowered to assess for a direct subgroup correlation between etiology and treatment response. All measures of myocardial deformation were improved in sinus rhythm patients, and circumferential strain and LVEF were considerably improved in sinus rhythm patients than in AF patients. This comparison is just hypothesis-generating, as previously stated. However, patients with AF showed a significantly improved peak longitudinal and radial strain after treatment, also highlighting the importance of sacubitril/valsartan therapy in this subgroup’s LV reverse remodeling. All LV tissue-tracking analyses performed showed an improvement in LV systolic deformation velocity by strain rate, while diastolic strain rate indices showed a specific improvement in the longitudinal direction. This considerable improvement in the LV myocardial deformation indices raises the possibility that reverse remodeling of the left heart may be a possible mechanism for generating improved cardiac outcomes in the PARADIGM-HF trial [1,34].

This is consistent with recent research which shows that sacubitril/valsartan therapy in HFrEF could induce a chronic and progressive LV unloading state that would relieve myocardial wall tensions and lower LV end-diastolic pressure, leading to cardiac reverse remodeling and restoration of the Frank–Starling mechanism of the LV myocardium, resulting in an increased LVEF [20]. However, although sacubitril/valsartan therapy improved LV systolic function in nonischemic and ischemic HFrEF etiology, the risk of ventricular tachyarrhythmias remains higher in ischemic HFrEF patients compared to nonischemic HFrEF patients [9].

Additionally, our findings suggest that STE parameters can be utilized as a helpful tool to assess the subclinical response to sacubitril/valsartan therapy and possibly serve as a guide for treatment in patients with HFrEF [35]. However, the reproducibility of these metrics to evaluate the improvement in LV contractility following a new HFrEF treatment may be hampered by the fact that LVEF [36] and myocardial strain indices [37] are relatively load dependent. Myocardial work, another newly developed dynamic STE tool, may also be helpful in determining treatment response [38].

### Study Limitations

It is important to recognize that our study has limitations. First of all, this was a prospective study that enrolled participants in a single center, and our findings need to be confirmed in more centers to be properly validated. Second, our study revealed encouraging outcomes on LA and LV strain after 6 months of therapy with sacubitril/valsartan, despite a small sample size. For a precise head-to-head comparison of the subgroups, specifically individuals with sinus rhythm versus individuals in AF and nonischemic HFrEF patients versus ischemic HFrEF patients, these findings would need be validated in larger trials. Thus, caution is required in the interpretation of our study’s results. Third, despite being a prospective study, the results were compared between baseline and after 6 months of sacubitril/valsartan therapy without a control group that would continue ACEI or ARB therapy. However, after the results of the PARADIGM-HF trial [1], it would not be ethical to withhold a treatment that had been shown to improve survival. A strategy to reduce bias by concomitant improvement caused by therapies other than sacubitril/valsartan was attempted by enrolling patients on optimized standard of care therapy (except for sacubitril/valsartan therapy), as detailed in Table 1, for more than 6 months and excluding patients with recent or planned major cardiovascular procedures (ICD or CRT implantation, coronary revascularization procedure, valvular treatment or catheter ablation of atrial fibrillation) which is demonstrated by no differences in beta-blockers and MRA dosage after 6 months of therapy, no new coronary revascularization procedures, valvular treatment or catheter ablation of atrial fibrillation and only one more patient with CRT-D at 6 months (implanted after an episode of third-degree atrioventricular block with syncope). Further studies should include a control group of patients continuing ACEI or ARB therapy for an accurate comparison with patients starting sacubitril/valsartan therapy. Importantly, in our sample only 31.4% of patients were taking SGLT2 inhibitors, as this drug class was not included in the criteria for optimal care medication for non-diabetic patients, according to the recommendations at the time of patient recruitment [24]. A larger proportion of patients using SGLT2 inhibitors should be included in future trials, given that the existing data show this class’s effects on reducing major cardiovascular events in HFrEF [39,40] and their impact on LV reverse remodeling [41,42]. The fact that the TTE scans were carried out by our center’s echocardiography staff, which were not blinded to the length of treatment, is another limitation of this study. In addition, conflicting data regarding a direct comparison of LA and LV strain analysis software across vendor platforms exist, as intervendor heterogeneity has been documented in strain analysis with speckle tracking [30,43,44]. Therefore, care should be used when comparing LA and LV strain on platforms from different suppliers, and the STE results from our study might not be applicable if analytic software from different vendors is employed.

## 5. Conclusions

In a small prospective trial, sacubitril/valsartan therapy in HFrEF patients was associated with improved cardiac performance as measured by LA phasic strain and strain rates as well as longitudinal, radial, and circumferential LV strain and strain rates, especially among patients in sinus rhythm. However, further studies are needed to investigate the impact of sacubitril/valsartan therapy among HFrEF patients. These findings may help in understanding the mechanisms underlying the improvement in cardiac function following sacubitril/valsartan therapy and gauge the treatment’s subclinical response.

## Figures and Tables

**Table 1:** Baseline characteristics of the study population (*n* = 35).

Characteristics	*n* (%)
Mean age (years)	58.6 ± 11.1
Male gender	29 (82.9%)
Ischemic etiology	15 (42.9%)
NYHA ≥ III	18 (51.4%)
Mean BMI (kg/m^2^)	28.1 ± 3.8
Heart failure hospitalization in the previous year	15 (42.9%)
Mean BNP (pg/mL)	375.3 ± 342.2
Current smoker	7 (20.0%)
Previous hypertension	25 (71.4%)
Dyslipidemia	25 (71.4%)
Diabetes mellitus	11 (31.4%)
Peripheral artery disease	4 (11.4%)
Family history of heart failure	1 (2.9%)
Atrial fibrillation	14 (40%)
Chronic kidney disease	2 (5.7%)
Chronic liver disease	0 (0)
Angiotensin-converting enzyme inhibitors	29 (82.9%)
Angiotensin II receptor blocker	6 (17.1%)
Beta-blocker	35 (100%)
Mineralocorticoid receptor antagonist	33 (94.3%)
Sodium-glucose co-transporter 2 inhibitor	11 (31.4%)
Ivabradine	13 (37.1%)
Digoxin	9 (25.7%)
Implantable cardioverter-defibrillator	30 (85.6%)
Cardiac resynchronization therapy	7 (20%)
Percutaneous mitral valve repair (TEER)	3 (8.6%)

NYHA, New York Heart Association; BMI, Body mass index; BNP, Natriuretic peptide B; TEER, Transcatheter Edge-to-Edge Repair.

**Table 2:** Echocardiographic data before and after 6 months of sacubitril/valsartan therapy.

Characteristics	Time 0	6 Months	*p*-Value
LV end-diastolic diameter (mm)	71.3 ± 8.4	66.9 ± 7.6	0.001
LV end-systolic diameter (mm)	57.8 ± 9.4	53.1 ± 9.3	0.002
Interventricular septum (mm)	9.6 ± 1.7	9.9 ± 1.9	0.280
LV ejection fraction (%)	29.3 ± 6.4	35.2 ± 8.6	0.001
Mean septal/lateral E/e’	13.6 ± 4.5	12.8 ± 4.6	0.449
Pulmonary artery systolic pressure (mmHg)	38.3 ± 12.2	30.9 ± 10.6	<0.001
Left atrium volume (mL/m^2^)	51.5 ± 22.6	43.7 ± 15.8	0.004
Right atrium volume (mL/m^2^)	33.1 ± 4.4	28.5 ± 13.5	0.036
Tricuspid annular systolic excursion (mm)	19.2 ± 4.4	20.0 ± 4.8	0.404
Left atrial strain parameters			
LA strain reservoir (%)	11.5 ± 6.2	16.1 ± 7.8	<0.001
LA strain conduit (%)	−6.3 [−8.5–−4.4]	−7.4 [−11.4–−5.0]	0.003
LA strain contraction (%) *	−7.2 ± 4.1	−10.9 ± 3.9	0.003
LA strain rate reservoir (s^−1^)	0.49 ± 0.22	0.65 ± 0.22	<0.001
LA strain rate conduit (s^−1^)	−0.47 [−0.68–−0.29]	−0.58 [−0.87–−0.39]	0.018
LA strain rate contraction (s^−1^) *	−0.82 [−1.14–−0.47]	−1.08 [−1.48–−0.94]	0.018
Left ventricular strain parameters			
Global longitudinal strain (%)	−7.0 ± 2.6	−8.9 ± 2.8	0.001
Peak longitudinal strain (%)	−5.6 ± 2.0	−9.3 ± 2.7	<0.001
Longitudinal systolic strain rate (s^−1^)	−0.32 ± 0.11	−0.47 ± 0.14	<0.001
Longitudinal early diastolic strain rate (s^−1^)	0.25 [0.18–0.41]	0.46 [0.27–0.62]	<0.001
Longitudinal late diastolic strain rate (s^−1^) *	0.31 ± 0.17	0.44 ± 0.19	0.002
Peak radial strain (%)	5.9 [4.3–9.2]	11.7 [7.7–14.7]	<0.001
Radial systolic strain rate (s^−1^)	0.66 ± 0.26	0.90 ± 0.30	0.001
Radial early diastolic strain rate (s^−1^)	−0.54 ± 0.48	−0.95 ± 0.72	0.001
Radial late diastolic strain rate (s^−1^) *	−0.58 [−0.86–−0.37]	−0.71 [−1.20–−0.51]	0.061
Peak circumferential strain (%)	−7.7 ± 2.3	−9.9 ± 2.5	0.001
Circumferential systolic strain rate (s^−1^)	−0.78 [−0.97–−0.58]	−0.87 [−1.10–−0.73]	0.026
Circumferential early diastolic strain rate (s^−1^)	0.87 ± 0.24	0.95 ± 0.21	0.102
Circumferential late diastolic strain rate (s^−1^) *	0.48 [0.34–0.78]	0.59 [0.50–0.82]	0.041

* Left atrial contraction phase strain, contraction strain rate and left ventricular late diastolic strain rates were assessed exclusively in patients in sinus rhythm during the echocardiogram (*n* = 26, 74.3%). LV, Left ventricle; LA, Left atrium, Values are mean ± SD or median [Q1–Q3].

**Table 3:** Echocardiographic parameters before and after 6 months of sacubitril/valsartan therapy according to sinus rhythm versus atrial fibrillation.

	Sinus Rhythm (*n* = 21)			Atrial Fibrillation (*n* = 14)			
Left Atrial Parameters	Time 0	6 Months	*p*-Value	Time 0	6 Months	*p*-Value	*p*-Value (Interaction)
LA volume (mL/m^2^)	43.8 ± 13.2	38.6 ± 13.4	0.041	67.6 ± 29.6	54.4 ± 15.3	0.051	0.147
Reservoir strain (%)	13.9 ± 5.9	19.8 ± 6.5	<0.001	7.0 ± 3.6	9.0 ± 4.5	0.080	0.027
Conduit strain (%)	−6.0 [−8.3–−4.4]	−7.3 [−10.8–−4.8]	0.024	−6.3 [−10.8–−3.9]	−7.4 [−14.4–−5.3]	0.056	0.850
Reservoir strain rate (s^−1^)	0.54 ± 0.20	0.74 ± 0.15	0.001	0.38 ± 0.23	0.46 ± 0.24	0.135	0.111
Conduit strain rate (s^−1^)	−0.49 [−0.74–−0.37]	−0.59 [−0.90–−0.41]	0.060	−0.34 [−0.60–−0.27]	−0.42 [−0.72–−0.34]	0.090	0.769
Left ventricular strain parameters							
LV end-diastolic diameter (mm)	70.8 ± 8.8	66.4 ± 7.4	0.006	72.6 ± 7.6	68.0 ± 8.4	0.052	0.975
LV ejection fraction (%)	29.6 ± 6.4	40.1 ± 7.9	<0.001	28.7 ± 6.5	33.9 ± 8.8	0.070	0.032
Global longitudinal strain (%)	−7.4 ± 2.5	−9.7 ± 2.5	0.001	−5.9 ± 2.7	−7.1 ± 2.7	0.227	0.299
Peak longitudinal strain (%)	−6.1 ± 2.0	−10.0 ± 2.6	<0.001	−4.5 ± 1.7	−7.8 ± 2.3	<0.001	0.477
Longitudinal systolic strain rate (s^−1^)	−0.32 ± 0.11	−0.50 ± 0.12	<0.001	−0.34 ± 0.12	−0.42 ± 0.17	0.095	0.046
Longitudinal early diastolic strain rate (s^−1^)	0.24 [0.17–0.41]	0.50 [0.27–0.65]	0.001	0.27 [0.20–0.41]	0.44 [0.25–0.52]	0.014	0.078
Peak radial strain (%)	5.9 [4.9–9.4]	11.3 [7.6–14.3]	0.001	5.2 [4.0–9.2]	12.9 [8.8–14.9]	0.005	0.618
Radial systolic strain rate (s^−1^)	0.68 ± 0.25	0.95 ± 0.30	0.002	0.63 ± 0.31	0.78 ± 0.19	0.227	0.357
Radial early diastolic strain rate (s^−1^)	−0.66 ± 0.31	−1.10 ± 0.62	0.005	−0.26 ± 0.67	−0.61 ± 0.85	0.117	0.739
Peak circumferential strain (%)	−7.5 ± 2.3	−10.7 ± 2.4	<0.001	−8.0 ± 2.4	−8.2 ± 1.7	0.855	0.028
Circumferential systolic strain rate (s^−1^)	−0.79 [−1.10–−0.62]	−0.98 [−1.25–−0.77]	0.009	−0.64 [−0.85–−0.50]	−0.67 [−0.74–−0.64]	0.919	0.129
Circumferential early diastolic strain rate (s^−1^)	0.84 ± 0.26	0.98 ± 0.23	0.025	0.90 ± 0.15	0.94 ± 0.17	0.563	0.065

LA, Left atrium; Values are mean ± SD or median [Q1–Q3].

**Table 4:** Echocardiographic parameters before and after 6 months of sacubitril/valsartan therapy according to nonischemic etiology versus ischemic etiology.

	Nonischemic Etiology (*n* = 20)			Ischemic Etiology (*n* = 15)			
Left Atrial Parameters	Time 0	6 Months	*p*-Value	Time 0	6 Months	*p*-Value	*p*-Value (Interaction)
LA volume (mL/m^2^)	56.2 ± 26.9	44.5 ± 15.8	0.005	44.9 ± 12.6	42.6 ± 16.2	0.442	0.053
Reservoir strain (%)	10.1 ± 4.8	15.0 ± 7.8	0.001	13.5 ± 7.4	17.7 ± 7.9	0.011	0.726
Conduit strain (%)	−5.5 [−9.5–−4.2]	−6.0 [−12.5–−4.2]	0.059	−6.4 [−8.5–−4.4]	−7.8 [−10.9–−6.9]	0.033	0.957
Contraction strain (%)	−6.2 ± 4.4	−10.6 ± 4.8	<0.001	−8.2 ± 3.6	−11.1 ± 2.7	0.052	0.327
Reservoir strain rate (s^−1^)	0.47 ± 0.21	0.65 ± 0.24	0.001	0.51 ± 0.24	0.64 ± 0.20	0.065	0.509
Conduit strain rate (s^−1^)	−0.52 [−0.79–−0.28]	−0.63 [−0.90–−0.35]	0.184	−0.46 [−0.63–−0.29]	−0.55 [−0.70–−0.41]	0.023	0.966
Contraction strain rate (s^−1^)	−0.58 [−1.10–0.33]	−1.22 [−1.51–−0.71]	0.016	−0.89 [−1.25–−0.66]	−1.07 [−1.28–−1.03]	0.285	0.096
Left ventricular strain parameters							
LV end-diastolic diameter (mm)	72.4 ± 7.9	66.6 ± 8.3	<0.001	70.0 ± 9.0	67.5 ± 6.9	0.225	0.167
LV ejection fraction (%)	28.1 ± 7.1	38.9 ± 10.2	<0.001	31.0 ± 5.0	37.2 ± 6.0	0.002	0.083
Global longitudinal strain (%)	−6.3 ± 2.1	−8.9 ± 3.3	0.003	−7.9 ± 3.0	−9.0 ± 2.1	0.092	0.146
Peak longitudinal strain (%)	−5.1 ± 1.6	−9.7 ± 3.1	<0.001	−6.2 ± 2.4	−8.8 ± 2.1	<0.001	0.007
Longitudinal systolic strain rate (s^−1^)	−0.31 ± 0.09	−0.50 ± 0.16	<0.001	−0.34 ± 0.14	−0.44 ± 0.12	0.003	0.054
Longitudinal early diastolic strain rate (s^−1^)	0.24 [0.17–0.39]	0.48 [0.29–0.62]	0.001	0.32 [0.18–0.42]	0.46 [0.27–0.65]	0.029	0.248
Longitudinal late diastolic strain rate (s^−1^)	−0.28 ± 0.16	−0.45 ± 0.23	0.031	0.34 ± 0.18	0.43 ± 0.16	0.029	0.371
Peak radial strain (%)	6.0 [4.3–9.8]	10.4 [6.7–15.6]	0.010	5.7 [4.8–7.9]	12.2 [10.0–13.9]	0.001	0.076
Radial systolic strain rate (s^−1^)	0.63 ± 0.23	0.95 ± 0.32	0.001	0.71 ± 0.31	0.83 ± 0.26	0.274	0.154
Radial early diastolic strain rate (s^−1^)	−0.44 ± 0.42	−0.90 ± 0.91	0.014	−0.67 ± 0.53	−1.03 ± 0.36	0.031	0.679
Radial late diastolic strain rate (s^−1^)	−0.50 [−0.97–−0.34]	−0.67 [−1.29–−0.51]	0.347	−0.66 [−0.84–−0.41]	−0.74 [−1.16–−0.45]	0.090	0.601
Peak circumferential strain (%)	−7.4 ± 2.5	−9.5 ± 2.7	0.036	−8.0 ± 2.0	−10.5 ± 2.2	0.008	0.779
Circumferential systolic strain rate (s^−1^)	−0.64 [−0.92–−0.51]	−0.87 [−1.08–−0.74]	0.021	−0.85 [−1.12–−0.71]	−0.87 [−1.13–−0.69]	0.530	0.135
Circumferential early diastolic strain rate (s^−1^)	0.88 ± 0.23	0.93 ± 0.22	0.396	0.87 ± 0.26	0.98 ± 0.21	0.081	0.598
Circumferential late diastolic strain rate (s^−1^)	0.45 [0.28–0.83]	0.65 [0.50–0.99]	0.170	0.51 [0.38–0.63]	0.58 [0.45–0.79]	0.099	0.609

LA, Left atrium; Values are mean ± SD or median [Q1–Q3].

## Data Availability

The data presented in this study are available on request from the corresponding authors. The data are not publicly available due to patient consent regarding availability of individual patient data, applicable only to the local investigation team.

## Supplementary Materials

The following supporting information can be downloaded at: https://www.mdpi.com/article/10.3390/life13040995/s1, Table S1: Baseline differences in echocardiographic parameters between patients in sinus rhythm versus patients in atrial fibrillation; Table S2: Baseline differences in echocardiographic parameters between patients with nonischemic etiology versus patients with ischemic etiology.
