# Supplementary material for: Sacubitril/Valsartan Improves Left Atrial and Ventricular Strain and Strain Rate in Patients with Heart Failure with Reduced Ejection Fraction
Source: Life (Basel). 2023 Apr 12;13(4):995. doi: 10.3390/life13040995 (PMC10142440; doi:10.3390/life13040995)
Supplement: Supplementary file 1 [file life-13-00995-s001.zip › Supplementary Table S1.docx]

**Supplementary Table S1.** Baseline differences in echocardiographic parameters between patients in sinus rhythm versus patients in atrial fibrillation

| **Left atrial strain parameters** | **Sinus rhythm (*n*=21)** | **Atrial fibrillation (*n*=14)** | ***p*-value** |
| --- | --- | --- | --- |
| LA volume (mL/m^2^) | 43.8 ± 13.2 | 67.6 ± 29.6 | 0.003 |
| LA strain reservoir (%) | 13.9 ± 5.9 | 7.0 ± 3.6 | <0.001 |
| LA strain conduit (%) | -6.0 [-8.3–-4.4] | -6.3 [-10.8–-3.9] | 0.665 |
| LA strain rate reservoir (s^-1^) | 0.54 ± 0.20 | 0.38 ± 0.23 | 0.043 |
| LA strain rate conduit (s^-1^) | -0.49 [-0.74–-0.37] | -0.34 [-0.60–-0.27] | 0.233 |
| **Left ventricular strain parameters** |  |  |  |
| End-diastolic diameter (mm) | 70.8 ± 8.8 | 72.6 ± 7.6 | 0.553 |
| Ejection fraction (%) | 29.6 ± 6.4 | 28.7 ± 6.5 | 0.719 |
| Global longitudinal strain (%) | -7.4 ± 2.5 | -5.9 ± 2.7 | 0.130 |
| Peak longitudinal strain (%) | -6.1 ± 2.0 | -4.5 ± 1.7 | 0.018 |
| Longitudinal systolic strain rate (s^-1^) | -0.32 ± 0.11 | -0.34 ± 0.12 | 0.657 |
| Longitudinal early diastolic strain rate (s^-1^) | 0.24 [0.17–0.41] | 0.27 [0.20–0.41] | 0.687 |
| Peak radial strain (%) | 5.9 [4.9–9.4] | 5.2 [4.0–9.2] | 0.499 |
| Radial systolic strain rate (s^-1^) | 0.68 ± 0.25 | 0.63 ± 0.31 | 0.659 |
| Radial early diastolic strain rate (s^-1^) | -0.66 ± 0.31 | -0.26 ± 0.67 | 0.021 |
| Peak circumferential strain (%) | -7.5 ± 2.3 | -8.0 ± 2.4 | 0.569 |
| Circumferential systolic strain rate (s^-1^) | -0.79 [-1.10–-0.62] | -0.64 [-0.85–-0.50] | 0.105 |
| Circumferential early diastolic strain rate (s^-1^) | 0.84 ± 0.26 | 0.90 ± 0.15 | 0.201 |

LA, Left atrium; Values are mean ± SD or median [Q1–Q3]
