# Supplementary material for: Sacubitril/Valsartan Improves Left Atrial and Ventricular Strain and Strain Rate in Patients with Heart Failure with Reduced Ejection Fraction
Source: Life (Basel). 2023 Apr 12;13(4):995. doi: 10.3390/life13040995 (PMC10142440; doi:10.3390/life13040995)
Supplement: Supplementary file 1 [file life-13-00995-s001.zip › Supplementary Table S2.docx]

**Supplementary Table S2.** Baseline differences in echocardiographic parameters between patients with nonischemic etiology versus patients with ischemic etiology

| **Left atrial strain parameters** | **Nonischemic (*n*=20)** | **Ischemic (*n*=15)** | ***p*-value** |
| --- | --- | --- | --- |
| LA volume (mL/m^2^) | 56.2 ± 26.9 | 44.9 ± 12.6 | 0.112 |
| LA strain reservoir (%) | 10.1 ± 4.8 | 13.5 ± 7.4 | 0.085 |
| LA strain conduit (%) | -5.5 [-9.5–-4.2] | -6.4 [-8.5–-4.4] | 0.747 |
| LA strain contraction (%) | -6.2 ± 4.4 | -8.2 ± 3.6 | 0.254 |
| LA strain rate reservoir (s^-1^) | 0.47 ± 0.21 | 0.51 ± 0.24 | 0.620 |
| LA strain rate conduit (s^-1^) | -0.52 [-0.79–-0.28] | -0.46 [-0.63–-0.29] | 0.679 |
| LA strain rate contraction (s^-1^) | -0.58 [-1.10–-0.33] | -0.89 [-1.25–-0.66] | 0.088 |
| **Left ventricular strain parameters** |  |  |  |
| End-diastolic diameter (mm) | 72.4 ± 7.9 | 70.0 ± 9.0 | 0.420 |
| Ejection fraction (%) | 28.1 ± 7.1 | 31.0 ± 5.0 | 0.160 |
| Global longitudinal strain (%) | -6.3 ± 2.1 | -7.9 ± 3.0 | 0.089 |
| Peak longitudinal strain (%) | -5.1 ± 1.6 | -6.2 ± 2.4 | 0.108 |
| Longitudinal systolic strain rate (s^-1^) | -0.31 ± 0.09 | -0.34 ± 0.14 | 0.434 |
| Longitudinal early diastolic strain rate (s^-1^) | 0.24 [0.17–0.39] | 0.32 [0.18–0.42] | 0.499 |
| Longitudinal late diastolic strain rate (s^-1^) | 0.28 ± 0.16 | 0.34 ± 0.18 | 0.427 |
| Peak radial strain (%) | 6.0 [4.3–9.8] | 5.7 [4.8–7.9] | 0.577 |
| Radial systolic strain rate (s^-1^) | 0.63 ± 0.23 | 0.71 ± 0.31 | 0.427 |
| Radial early diastolic strain rate (s^-1^) | -0.44 ± 0.42 | -0.67 ± 0.53 | 0.174 |
| Radial late diastolic strain rate (s^-1^) | -0.50 [-0.97–-0.34] | -0.66 [-0.84–-0.41] | 0.810 |
| Peak circumferential strain (%) | -7.4 ± 2.5 | -8.0 ± 2.0 | 0.450 |
| Circumferential systolic strain rate (s^-1^) | -0.64 [-0.92–-0.51] | -0.85 [-1.12–-0.71] | 0.098 |
| Circumferential early diastolic strain rate (s^-1^) | 0.88 ± 0.23 | 0.87 ± 0.26 | 0.971 |
| Circumferential late diastolic strain rate (s^-1^) | 0.45 [0.28–0.83] | 0.51 [0.38–0.63] | 0.746 |

LA, Left atrium; Values are mean ± SD or median [Q1–Q3]
